# Recombinant Human Dentin Matrix Protein 1 (hDMP1) Expressed in *Nicotiana benthamiana* Potentially Induces Osteogenic Differentiation

**DOI:** 10.3390/plants8120566

**Published:** 2019-12-03

**Authors:** Aktsar Roskiana Ahmad, Pornjira Kaewpungsup, Narach Khorattanakulchai, Kaewta Rattanapisit, Prasit Pavasant, Waranyoo Phoolcharoen

**Affiliations:** 1 Department of Pharmacognosy and Pharmaceutical Botany, Faculty of Pharmaceutical Sciences, Chulalongkorn University, Bangkok 10330, Thailand; aktsar.roskiana@umi.ac.id; 2 Department of Pharmacognosy and Phytochemistry, Faculty of Pharmacy, Universitas Muslim Indonesia, Makassar 90231, Indonesia; 3 Research Unit of Mineralized Tissue, Faculty of Dentistry, Chulalongkorn University, Bangkok 10330, Thailand; pornjira.k@ku.th; 4 Research Unit for Plant-produced Pharmaceuticals, Chulalongkorn University, Bangkok 10330, Thailand; 6271012833@student.chula.ac.th (N.K.); kaewta.R@chula.ac.th (K.R.); 5 Department of Anatomy, Faculty of Dentistry, Chulalongkorn University, Bangkok 10330, Thailand

**Keywords:** human dentin matrix protein-1 (hDMP1), *Nicotiana benthamiana*, plant-produced recombinant protein, molecular pharming, transient expression, osteogenic differentiation

## Abstract

Inductive molecules are critical components for successful bone tissue engineering. Dentin matrix protein-1 (DMP1), a non-collagenous protein in the bone matrix, has been shown to play roles in osteogenic differentiation and phosphate homeostasis. This study aimed to produce recombinant human dentin matrix protein-1 (hDMP1) in *Nicotiana benthamiana* and investigated the ability of this plant-produced DMP1 to induce osteogenesis in human periodontal ligament stem cells (hPDLSCs). The hDMP1 gene was cloned into the geminiviral vector for transient expression in *N. benthamiana.* We found that hDMP1 was transiently expressed in *N. benthamiana* leaves and could be purified by ammonium sulphate precipitation followed by nickel affinity chromatography. The effects of hDMP1 on the induction of cell proliferation and osteogenic differentiation were investigated. The results indicated that plant-produced hDMP1 could induce the cell proliferation of hPDLSCs and increase the expression levels of osteogenic genes, including osterix (OSX), type I collagen (COL1), bone morphogenetic protein-2 (BMP2), and Wnt3a. Moreover, the plant-produced hDMP1 promoted calcium deposition in hPDLSCs as determined by alizarin red S staining. In conclusion, our results indicated that plant-produced hDMP1 could induce osteogenic differentiation in hPDLSCs and could potentially be used as a bone inducer in bone tissue engineering.

## 1. Introduction

Tissue engineering is one of the clinical therapeutic strategies used to address bone defects. Currently, methods to promote regeneration after bone damage involve the use of bone grafts (autologous or allogeneic) or polymeric bone scaffolds [1]. However, these approaches still have some limitations, such as disease transmission, high cost, and the inability to incorporate into the surrounding host tissue [2]. Therefore, approaches that seek to overcome these limitations are necessary. The use of biomolecules, such as matrix proteins or growth factors that show osteogenic differentiation potential, can induce the appropriate signaling pathways to promote bone regeneration [3]. In scaffold-based tissue engineering, a suitable surface to promote bone cell adhesion and differentiation could enhance the success of bone regeneration. It has been shown that some extracellular matrix (ECM) proteins, such as collagen I and laminin-5, can stimulate osteogenic differentiation of mesenchymal stem cells by MAPK activation [4,5]. Dentin matrix protein 1 ( DMP1) is a component of the bone matrix that plays a role in bone development [6]. Moreover, DMP1 contains an RGD sequence in its structure that supports cell adhesion via integrins [7]. Therefore, dentin matrix protein 1 (DMP1) is a good candidate for inducing bone differentiation.

DMP1 is a non-collagenous ECM component that has acidic properties. DMP1 plays a critical role in osteogenic differentiation and phosphate homeostasis in osteoblast/osteocyte lineage cells [8,9,10,11]. Recombinant DMP1 protein has been produced in *Escherichia coli*, yeast, insect, and mammalian cells. In our study, we aimed to develop recombinant DMP1 protein in plants. Plants have several advantages over other systems, such as post-translational modifications, low production cost, and scalability [12]. In addition, adventitious agents are absent in plants. Therefore, using a plant platform can decrease the risk of pathogen contamination [13]. Several plants, such as alfalfa, soybean, lettuce, potato, spinach, *Arabidopsis*, and tobacco, have been used to produce recombinant proteins [12,13,14,15]. Among these plants, tobacco has several benefits, such as well-established transformation protocols, high biomass, scale-up capacity, its role as a non-food crop, prolific seed production, and year-round growth. *Nicotiana benthamiana* is a common species of tobacco that is used in transient expression studies [16].

In this study, we produced the recombinant protein human dentin matrix protein 1 (hDMP1) in *N. benthamiana* and tested the effect of this protein on the proliferation, osteogenic differentiation, and calcification deposition of human periodontal ligament stem cells (hPDLSCs) Based on these results, plant-produced hDMP1 is a promising candidate for a **signaling** molecule in tissue engineering.

## 2. Results

### 2.1. Optimized Expression of Human Dentin Matrix Protein 1 (hDMP1) in N. benthamiana

**Figure 1:**
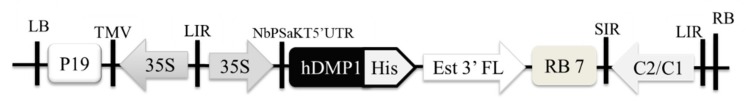
Schematic representation of the T-DNA regions in the geminiviral vector used in this study; 35S: cauliflower mosaic virus 35S promoter; hDMP1: human dentin matrix protein 1 gene with 8X histidine residues at the C-terminus; Est 3’ FL: expressed sequence tags-full length at the 3’ end of transcription; Rb 7: tobacco RB7 promoter; C2/C1: bean yellow dwarf virus (BeYDV) ORFs C1 and C2, which encode the replication initiation protein (Rep) and RepA; LIR: long intergenic region of the BeYDV genome; SIR: short intergenic region of the BeYDV genome; P19: P19 gene from tomato bushy stunt virus (TBSV); LB: the left border, RB: the right border.

**Figure 2:**
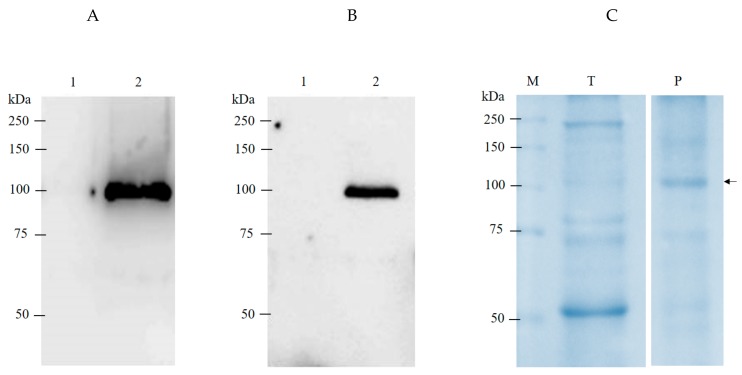
The expression of human dentin matrix protein-1 (hDMP1) in *Nicotiana benthamiana*. Western blot was probed with anti-His antibody (**A**) or anti-human DMP1 antibody (**B**). Lane 1: non-infiltrated plant crude extract; Lane 2: infiltrated crude extract. (**C**) The coomassie staining of plant crude extract and purified hDMP1. M: protein marker; T: total soluble protein from infiltrated plant; P: purified hDMP1.

The hDMP1 gene was optimized in silico with an *N. benthamiana-*optimized codon, cloned into geminiviral vectors (Figure 1) and expressed in *N. benthamiana* leaves. The plasmid was introduced to *Agrobacterium tumefaciens* strain GV3101 and infiltrated into *N. benthamiana* leaves. After that, the hDMP1 protein in plant extracts was detected by Western blot analysis using anti-His and anti-DMP1 antisera (Figure 2). The *enzyme-linked immunosorbent assay* (ELISA) result showed that the highest expression level of hDMP1 in *N. benthamiana* leaves occurred on day two post-infiltration with OD_600_ Agrobacterium 0.4. at the amount of 0.3 µg/g fresh weight. The hDMP1 protein was purified by ammonium sulphate precipitation followed by Ni affinity column.

### 2.2. Effect of hDMP1 on Cell Proliferation

**Figure 3:**
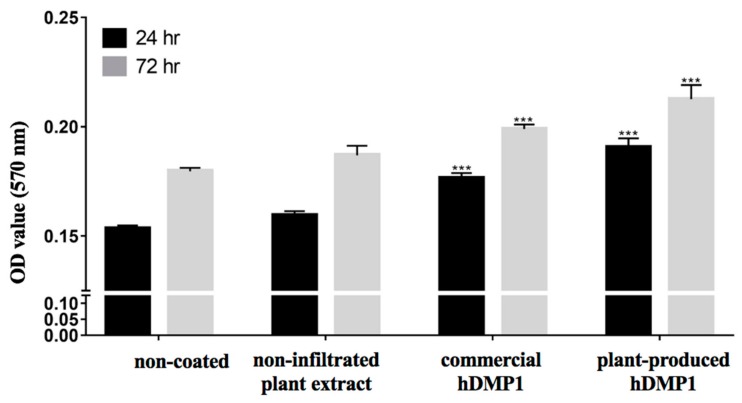
Effect of hDMP1 on human periodontal ligament stem cell (hPDLSC) proliferation. Three lines of hPDLSCs were collected from three different donors. The hPDLSCs were cultured on plates coated with 2 µg/mL of plant-produced hDMP1, commercial hDMP1, or non-infiltrated plant extract and a non-coated plate for 24 and 72 h before the MTT assay was performed. Data represent the absorbance at 570 nm. (*** indicates a significant difference compared to the cells growing on the non-coated plate, ***: *p* < 0.001).

The effect of plant-produced hDMP1 on cell proliferation was examined by MTT assays, and the results are shown in Figure 3. Human periodontal ligament stem cells (hPDLSCs) were seeded on culture plates coated with 2 µg/mL plant-produced hDMP1, 2 µg/mL commercial hDMP1 (R&D Systems, USA), or non-infiltrated plant extract. The non-coated wells were used as the negative control. Both plant-produced hDMP1 and commercial DMP1 significantly increased the proliferation of hPDLSCs compared to those on the non-coated and plant-extract-coated surfaces.

### 2.3. Plant-Produced hDMP1 Activates Osteogenesis-Related Genes

**Figure 4:**
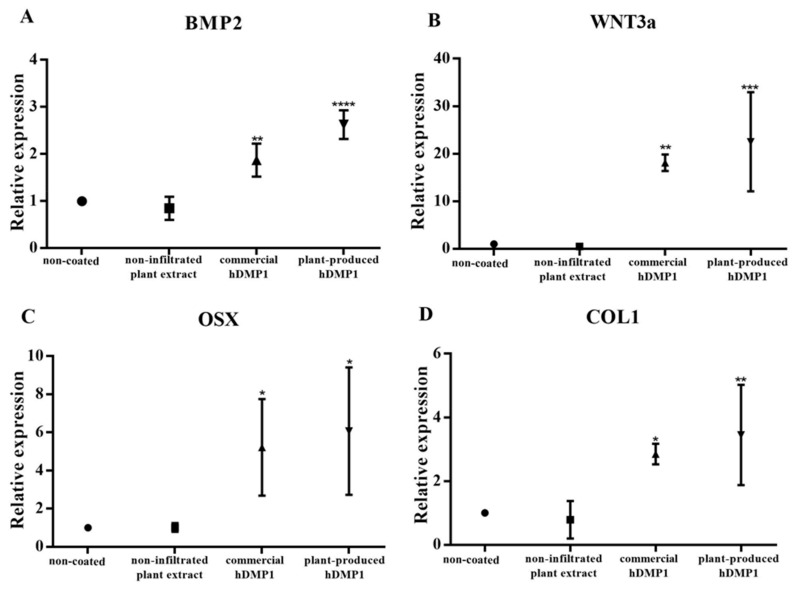
Plant-produced DMP1 increased the mRNA expression of osteogenic-genes. Four lines of the hPDLSCs were collected from three different donors. The hPDLSCs were cultured on plates coated with plant-produced hDMP1, commercial hDMP1, and plant extract and non-coated plates for 3 days. The mRNA was extracted, and RT-PCR was performed to analyze the expression of BMP2 (**A**), WNT3a (**B**), OSX (**C**), and COL1 (**D**). The hPDLSCs from four different donors were treated with 2 µg/mL plant-produced hDMP1, commercial hDMP1 (R&D Systems, Minneapolis, MN, USA), or plant extract, or grown on a non-coated plate. (* indicates a significant difference compared to the cells growing on a non-coated plate, *: *p* < 0.05, **: *p* < 0.01, ***: *p* < 0.001 and ****: *p* < 0.0001).

The expression levels of osteogenic genes, including osterix (OSX), type I collagen (COL1), bone morphogenetic protein-2 (BMP2), and Wnt3a, were examined using real-time PCR. Human periodontal ligament stem cells (hPDLSCs) were cultured on culture plates coated with 2 µg/mL plant-produced hDMP1, commercial hDMP1 (R&D Systems, USA), or plant extract and non-coated plates for three days. The mRNA was extracted and subjected to RT-PCR analysis to examine the *BMP2, WNT3a, OSX*, and *COL1* gene expression. Figure 4 shows the expression levels of these genes compared to those from cells cultured on the non-coated surface or the surface coated with plant extract. The results indicated that both commercial and plant-produced DMP1 proteins could significantly induce the expression of the *BMP2, WNT3a, OSX*, and *COL1* genes.

### 2.4. Plant-Produced hDMP1 Activates Calcification

**Figure 5:**
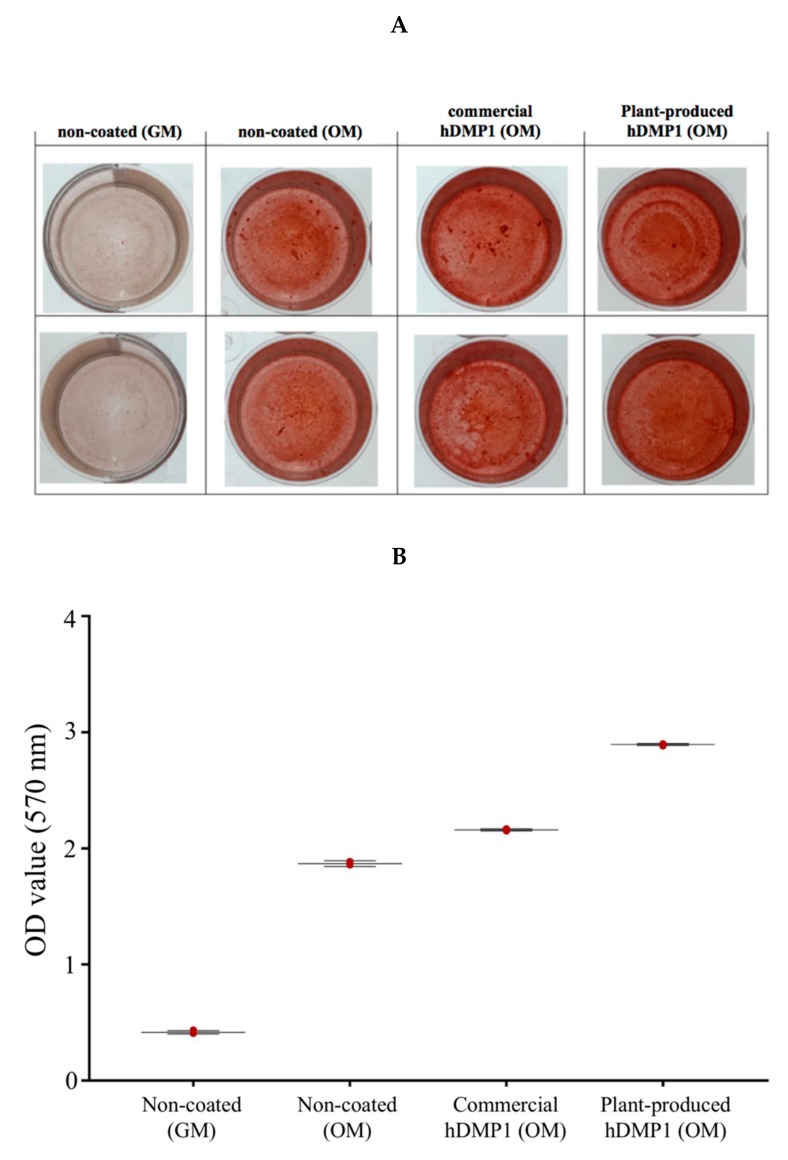
Calcification assay of the hPDLSCs. The cells were treated with 2 µg/mL plant-produced hDMP1 and commercial hDMP1 and then cultured in osteogenic medium (OM) and general medium (GM) as a control for 14 days. The findings show that plant-produced hDMP1 at 2 µg/mL could increase the calcification in the hPDLSCs (**A**), which was confirmed by quantification of Alizarin red S staining (**B**).

The effect of plant-produced DMP1 on the in vitro calcification was examined. Cells were cultured on the surface coated with either commercial or plant-produced hDMP1 in osteogenic medium (OM). The culture in normal growth medium (GM) was used as a negative control. The results showed that hPDLSCs cultured on uncoated culture vessels in the OM, but not the GM, for 14 days displayed calcium deposition or in vitro calcification, which could be seen as positive alizarin red S staining (Figure 5A). Interestingly, in cells that were cultured on both commercial DMP1 or plant-produced hDMP1, enhanced in vitro calcification was observed (Figure 5A). Quantitative analysis from triplicate cultures using cells from three different donors is shown as a graph in Figure 5B. The results confirmed that plant-produced hDMP1 significantly induced in vitro calcification in hPDLSCs.

## 3. Discussion

Among several recombinant protein production platforms, plants have become an attractive option due to various advantages, such as low production costs, the lack of human and animal pathogens, post-translational modification, and scalability [17]. Plants have been used to produce a variety of recombinant proteins, such as monoclonal antibodies, vaccines, therapeutic proteins, enzymes, etc. [18,19,20,21].

Previous studies have demonstrated the production of recombinant DMP1 in *E. coli* and wheat germ [22,23,24]. However, the expression level of this protein in a different host varies. In this study, recombinant human dentin matrix protein-1 (hDMP1) was transiently expressed in *N. benthamiana* leaves. We optimized the expression of hDMP1 in plants, and the protein was expressed rapidly. The highest expression was detected on day two post-infiltration with *Agrobacterium*. The expected size of full-length DMP1 is approximately 55 kDa without any post-translational modifications [25]. Previous studies have demonstrated the dimerization of the DMP1 protein, resulting in a size of approximately 100 kDa [22]. The expression of recombinant hDMP1 with a histidine tag in crude protein was confirmed by probing with anti-histidine and anti-hDMP1 antibodies and was detected at approximately 100 kDa under reducing conditions (Figure 2). This finding confirms the extensive dimerization of plant-produced DMP1. However, previous studies have reported that modification of rat DMP1 could increase the size of this molecule to 160–210 kDa, as indicated by immunoblotting analysis with anti-DMP1 antibody [26]. In addition, mouse DMP1 was analyzed via Western blots using an anti-mouse DMP1 antibody and showed a size of 130–150 kDa. Subsequently, recombinant mouse DMP1 expressed in wheat germ or *E. coli* had a size of approximately 110 kDa [24]. Post-translational modifications might cause the different molecular weights of DMP1 proteins from different sources, which might increase the molecular weights of the proteins [24].

After Ni affinity chromatography, purified hDMP1 was tested for its effect on the proliferation of human periodontal ligament stem cells (hPDLSCs) by MTT assays. We performed the MTT assays at 24 and 72 h. In all studies, we compared plant-produced hDMP1 with commercial hDMP1 produced in a mouse myeloma cell line (R&D Systems, USA). The results showed that both types of proteins could significantly increase the number of hPDLSCs cells at both time points, which also indicated that the plant-produced proteins were not cytotoxic. However, hPDLSCs treated with plant-produced hDMP1 showed the highest survival rate (Figure 3).

DMP1 is expressed in osteoblasts and odontoblasts [8,27,28]. DMP1 was shown to activate the transcription of osteoblast and odontoblast gene markers, such as runt-related transcription factor 2 (Runx2), in the nucleus [27,28]. Consistent with these reports, the results from this study also showed that DMP-1 could induce osterix (OSX), a key transcription factor for osteogenic differentiation, as well as COL-I (type I Collagen), the major component of bone and dentin matrix (Figure 4). We also found that DMP-1 could induce the expression of BMP2 and Wnt3a, two signaling molecules involved in the regulation of osteogenic differentiation of hPDLSCs (Figure 4). Human periodontal ligament cells have been shown to possess mesenchymal stem cell characteristics and play important roles in the repair and regeneration of periodontal tissue, including alveolar bone [29,30]. The inductive effects of the plant-produced DMP1 were comparable to the effects of commercial hDMP1 produced in a mouse myeloma cell line (R&D Systems, USA), supporting the concept that plant-produced protein can influence cellular behavior similar to the protein produced from mammalian cells.

Moreover, our results showed that *N. benthamiana*-produced hDMP1 resulted in higher levels of calcium deposition than commercial hDMP1 (Figure 5). These results not only confirmed the inductive effect of DMP-1 but also indicated the potential of *N. benthamiana*-produced hDMP1 as a new candidate for tissue engineering via induction of osteogenic differentiation.

The results from this study showed that hDMP1 was successfully produced in *N. benthamiana* and could induce osteoblast differentiation. However, this study did not describe the detailed mechanisms. The molecular mechanism of recombinant hDMP1 in osteogenic differentiation is unclear. Overall, our study demonstrated that plant-produced hDMP1 could induce proliferation, osteogenic differentiation, and calcium deposition of hPDLSCs. The plant platform has the potential to produce recombinant proteins for developing tissue engineering technology in the future.

## 4. Materials and Methods

### 4.1. Construction of pBYR2e-hDMP1

The plant-optimized DNA sequence encoding human DMP1 was based on a previous study [22]. The gene encoding hDMP1 with 8xHis was inserted into the geminiviral expression vector pBYR2e [31] by using the *Xba*I and *Sac*I restriction enzyme (New England BioLabs, Ipswich, MA, USA). The pBYR2e-DMP1 vector (Figure 1) was transformed into *E. coli* DH10B by using the heat shock method. The selected colonies were analyzed by colony PCR and cultured in Luria Bertani (LB) media (HiMedia Laboratories, Mumbai, India) with 100 mg/mL ampicillin (ITW Reagents, Darmstadt, Germany) at 37 °C overnight.

### 4.2. Expression of hDMP1 in N. benthamiana

pBYR2e-hDMP1 was transformed into *A. tumefaciens* GV3101 by electroporation. The colonies that contained gene insertions were verified via PCR. Then, the bacteria were grown in LB media supplemented with 50 µg/mL kanamycin (Bio Basic, Markham, ON, Canada), 50 µg/mL gentamicin (ITW Reagents, Darmstadt, Germany) and 50 µg/mL rifampicin (Thermo Fisher Scientific, Waltham, MA, USA) overnight at 28 °C. Briefly, after centrifugation (15 min at 4000× *g*), pelleted bacteria were collected and resuspended in infiltration buffer (100 mM 2-(N-morpholino) ethanesulfonic acid (MES) and 100 mM MgSO_4_, pH 5.5). The solution was used for infiltrating *N. benthamiana* (6–8-week-old) leaves. To optimize the expression level of hDMP1 in *N. benthamiana*, different concentrations of *Agrobacterium* (OD_600_ 0.1, 0.2, 0.3, 0.4, 0.5 and 1.0) were infiltrated, and the leaves were harvested on days 1, 2, 3, 4, and 5 post-infiltration.

### 4.3. Purification of hDMP1

The *N. benthamiana* leaves were extracted with extraction buffer (20 mM Tris pH 7.4, 50 mM NaCl, and 5 mM imidazole) and then centrifuged at 26,000 rpm for 30 min. RuBisCO was removed from the protein extract via ammonium sulphate precipitation. Ammonium sulphate was added to the solution to 35% saturation, stirred for 30 min on ice, and then centrifuged at 4000 g for 30 min. The supernatant was added to 80% ammonium sulphate saturation, stirred for 30 min and centrifuged at 4000× *g* for 30 min. Then, the pellet was resuspended with extraction buffer and filtered by using a 0.45 µm syringe filter (Merck, Cork, Ireland). The sample was loaded onto a Ni-NTA affinity column (Qiagen Gmbh, Hilden, Germany). Further, the column was washed with washing buffer (20 mM Tris pH 7.4, 50 mM NaCl, and 20 mM imidazole) and eluted with elution buffer (20 mM Tris pH 7.4, 50 mM NaCl, and 250 mM imidazole).

### 4.4. SDS PAGE and Western Blot

The samples were suspended in 10X reducing SDS loading dye buffer (125 mM Tris HCl, 12% SDS, 10% glycerol, 22% β-mercaptoethanol, 0.001% bromophenol blue, pH 6.8). The proteins were separated by using 10% SDS-PAGE and detected by coomassie staining and Western blot analysis. The gel was subsequently stained with Coomassie Brilliant Blue R250 (ITW Reagents, Darmstadt, Germany) solution (in methanol: H2O (1:1)) overnight. The gel was transferred into a nitrocellulose membrane (Thermo Fisher Scientific, Waltham, MA, USA). Then, the membrane was probed with HRP-conjugated goat anti-His antibodies (Abcam, Cambridge, UK) and rabbit anti-human DMP1 antibodies (Abcam, Cambridge, UK) diluted at 1:5000 in 3% skim milk in 1× TBS and HRP-conjugated goat anti-rabbit IgG antibodies (Jackson ImmunoResearch, USA) diluted at 1:5000 in 3% skim milk in 1× TBS. The protein band was detected with an enhanced chemiluminescence (ECL) (GE Healthcare Life Sciences, Pittsburgh, PA, USA) reagent.

### 4.5. DMP1 Quantification by Enzyme-Linked Immunosorbent Assays

The DMP1 protein was quantified by enzyme-linked immunosorbent assays (ELISAs). The procedure was based on the manual of the hDMP1 ELISA Kit (Thermo Fisher Scientific, Waltham, MA, USA).

### 4.6. Cell Culture

Periodontal ligament cells were established from periodontal ligament tissue obtained from impacted lower molar extracted for orthodontic reasons at the Department of Surgery, Faculty of Dentistry, Chulalongkorn University. This study was approved by the human subject ethics board of the Faculty of Dentistry, Chulalongkorn University and was conducted following the Helsinki Declaration of 1975, as revised in 2013. Human periodontal ligament stem cells (hPDLSCs) were isolated and cultured according to previous reports [32]. In brief, the periodontal ligament was scraped from the middle of the root and placed in a culture vessel for 3–4 weeks in high glucose-Dulbecco’s modified Eagle’s medium (DMEM) containing 10% foetal bovine serum, 1% L-glutamine (2 mM), penicillin (100 U/mL), streptomycin (100 mg/mL), and amphotericin B (5 mg/mL). The cells were maintained in a humidified atmosphere of 5% CO_2_ at 37 °C. After the cells reached confluency, they were detached with 0.25% trypsin-EDTA and subcultured at a ratio of 1:3. Cells from the third to the fifth passages were used in the experiments.

### 4.7. The DMP1 Coating Protocol

The wells were coated with dopamine for 6 h. Next, the cells were washed with PBS buffer and coated with plant-produced hDMP1 (2 µg/mL) and commercial hDMP1 (2 µg/mL) overnight. Then, as many as 50,000 hPDLSCs were seeded into each well. The hPDLSCs were incubated.

### 4.8. Cell Proliferation Assay

Proliferation analyses were performed to determine the cell vitality using MTT assays (3-[4,5-dimethylthiazole-2-yl]-2,5-diphenyltetrazolium bromide) (USB Corporation, Cleveland, OH, USA). The hPDLSCs were collected from three different donors. Each well (24-well plate) was seeded with 50,000 cells. The hPDLSCs were cultured on *N. benthamiana-*produced hDMP1 (2 µg/mL), commercial hDMP1 (2 µg/mL) (R&D Systems, USA), and WT (1 µg/mL) for 24 and 72 h. Then, as much as 500 μL of MTT reagent with a concentration of 0.5 mg/mL was added to every well and incubated at 37 °C for 30 min. Elution with 500 µL of glycine buffer (pH 10, 100 mM NaCl and 100 mM glycine: DMSO (1:9)) was performed. The absorbance was measured using a spectrophotometer at a wavelength of 570 nm on a universal microplate reader (Bio-Tek Instruments, Inc., Winooski, VT, USA). All experiments were performed in triplicate. Statistical analysis was performed using GraphPad 7 and one-way ANOVA.

### 4.9. Real-Time PCR (RT-PCR) Analysis

The hPDLSCs were cultured on the plates with *N. benthamiana-*produced hDMP1 (2 µg/mL) and commercial hDMP1 (2 µg/mL) (R&D Systems, Minneapolis, MN, USA) for 3 days of incubation. The mRNA of hPDLSCs was extracted and used to generate cDNA by using a reverse transcriptase kit (Promega, Madison, WI, USA). The RT-PCR analysis was performed on a Light Cycler Nano system (Roche Applied Science, Indianapolis, IN, USA) with Fast Start Essential DNA Green Master (Roche Applied Science, Penzberg, Germany). The primers for (Table 1) OPN, OSX, BMP2, and WNT3a were used for this experiment. The RT-PCR conditions were as follows: denaturation at 94 °C for 10 min, annealing at 60 °C for 10 sec, and extension at 72 °C for 10 sec for 45 cycles. The experiments were performed with four replicates. Statistical analysis was performed using Graph Pad 7 and one-way ANOVA.

### 4.10. Calcium Deposition

The plant-produced hDMP1 (2 µg/mL), commercial-hDMP1 (2 µg/mL) (R&D Systems, USA), and WT were incubated in the wells overnight. Then, hPDLSCs were seeded at 50,000 cells per well in serum-free medium (SFM) and incubated for 6 h. Furthermore, the media were changed to osteogenic medium (10% DMEM supplemented with 100 nM dexamethasone, (50 mg/mL) ascorbic acid, and 10 mM b-glycerophosphate) and general medium (10% DMEM, 10% FBS, 1% GlutaMAX, 1% antibiotic-antimitotic) as a control and then incubated for 14 days. Next, phosphate-buffered saline (PBS) was used for washing the cells, and then, they were fixed with cold methanol for 10 min. Eventually, staining was performed using 1% alizarin red S solution (Sigma-Aldrich, St. Louis, MO, USA) followed by a wash with deionized water, and then, the samples were dried at room temperature. Finally, quantification was performed with 10% cetylperidium chloride in aqueous 10 mM sodium phosphate solution at pH 7, and then, the absorbance was measured on a microplate reader at 570 nm.

## 5. Conclusions

The hDMP1 protein can transiently be expressed in *N. benthamiana* leaves. The highest level of hDMP1 was obtained at two days after infiltration and OD_600_ 0.4. The plant-produced hDMP1 not only significantly induces the osteogenic genes *OSX, COL1, BMP2*, and *WNT3a* but also stimulates calcium deposition in hPDLSCs.

## Figures and Tables

**Table 1:** Primer sequences for gene expression analysis using RT-PCR.

Gene	Sequence	Reference
OSX	F; 5′ GCCAGAAGCTGTGAAACCTC 3′ R; 5′ GCTGCAAGCTCTCCATAACC 3′	NM-001300837.1
OPN	F; 5′ AGGAGGAGGCAGAGCACA 3′ R; 5′ CTGGTATGGCACAGGTGATG 3′	NM-001040060.1
BMP2	F; 5′ CTCAGCGAGTTTGAGTTGAGG 3′ R; 5′ GGTACAGGTCGAGCATATAGGG 3′	NM-017178.1
WNT3a	F; 5′ CTGTTGGGCCACAGTATTCC 3′ R; 5′ GGGCATGATCTCCACGTAGT 3′	NM-033131.3
